# Pair distribution function analysis of sulfide glassy electrolytes for all-solid-state batteries: Understanding the improvement of ionic conductivity under annealing condition

**DOI:** 10.1038/s41598-017-07086-y

**Published:** 2017-08-01

**Authors:** Shinya Shiotani, Koji Ohara, Hirofumi Tsukasaki, Shigeo Mori, Ryoji Kanno

**Affiliations:** 1 Battery Research Department, Toyota Motor Corporation, Shizuoka, 410-1193 Japan; 2 Research and Utilization Division, Japan Synchrotron Radiation Research Institute (JASRI, SPring-8), Hyogo, 679-5198 Japan; 3 Department of Materials Science, Osaka Prefecture University, Osaka, 599-8531 Japan; 4 Department of Chemical Science and Engineering, Tokyo Institute of Technology, Kanagawa, 226-8502 Japan

## Abstract

In general, the ionic conductivity of sulfide glasses decreases with their crystallization, although it increases for a few sulphide glasses owing to the crystallization of a highly conductive new phase (e.g., Li_7_P_3_S_11_: 70Li_2_S-30P_2_S_5_). We found that the ionic conductivity of 75Li_2_S-25P_2_S_5_ sulfide glass, which consists of glassy and crystalline phases, is improved by optimizing the conditions of the heat treatment, i.e., annealing. A different mechanism of high ionic conductivity from the conventional mechanism is expected in the glassy phase. Here, we report the glassy structure of 75Li_2_S-25P_2_S_5_ immediately before the crystallization by using the differential pair distribution function (d-PDF) analysis of high-energy X-ray diffraction. Even though the ionic conductivity increases during the optimum annealing, the d-PDF analysis indicated that the glassy structure undergoes no structural change in the sulfide glass-ceramic electrolyte at a crystallinity of 33.1%. We observed the formation of a nanocrystalline phase in the X-ray and electron diffraction patterns before the crystallization, which means that Bragg peaks were deformed. Thus, the ionic conductivity in the mixture of glassy and crystalline phases is improved by the coexistence of the nanocrystalline phase.

## Introduction

Sulfide glass ceramics are of interest for use as solid electrolytes in lithium ion batteries^1^, because the realization of an all-solid-state battery will enable the miniaturization of battery packages and reduce safety issues. Significant progress has been made so far with the discovery of numerous sulfide compounds with high ionic conductivities such as Li_7_P_3_S_11_^2, 3^, Li_10_GeP_2_S_12_^4, 5^, Li_7_P_2_S_8_I^6^, Li_10_SnP_2_S_12_^7^, and 90Li_7_P_3_S_11_ -10LiBr^8^. Their conductivities are higher than those of the corresponding sulfide glasses. In general, the crystallization of glassy materials results in a decrease in the conductivity^9^, although it increases in a few sulfide glasses owing to the crystallization of a highly conductive new phase^2^. Recently, we reported that 75Li_2_S-25P_2_S_5_ glass in the binary Li_2_S-P_2_S_5_ system with strongly polarized sulfur has high ionic conductivity^10^. In the sulfide glasses as below reported here, an interesting improvement in the conductivity is observed during annealing, which appears in the glassy phase. In this paper, we report the local glassy structure with a mixture of glass and crystalline phases by using pair distribution function (PDF) analysis.

## Results and Discussion

**Figure 1:**
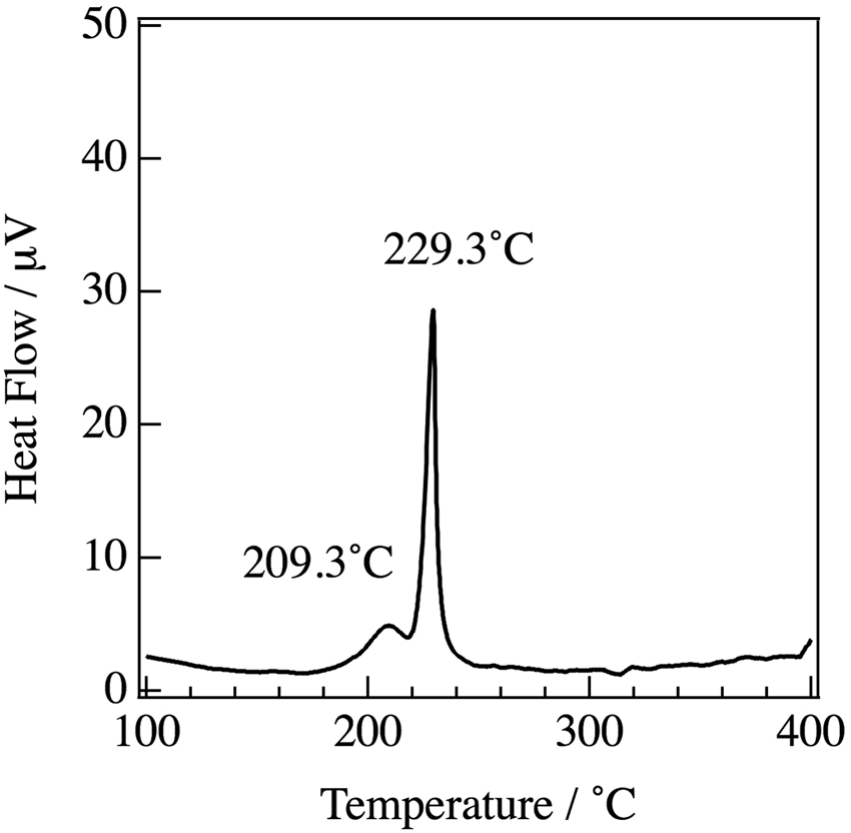
DTA curve for the 75Li_2_S-25P_2_S_5_ glass.

**Figure 2:**
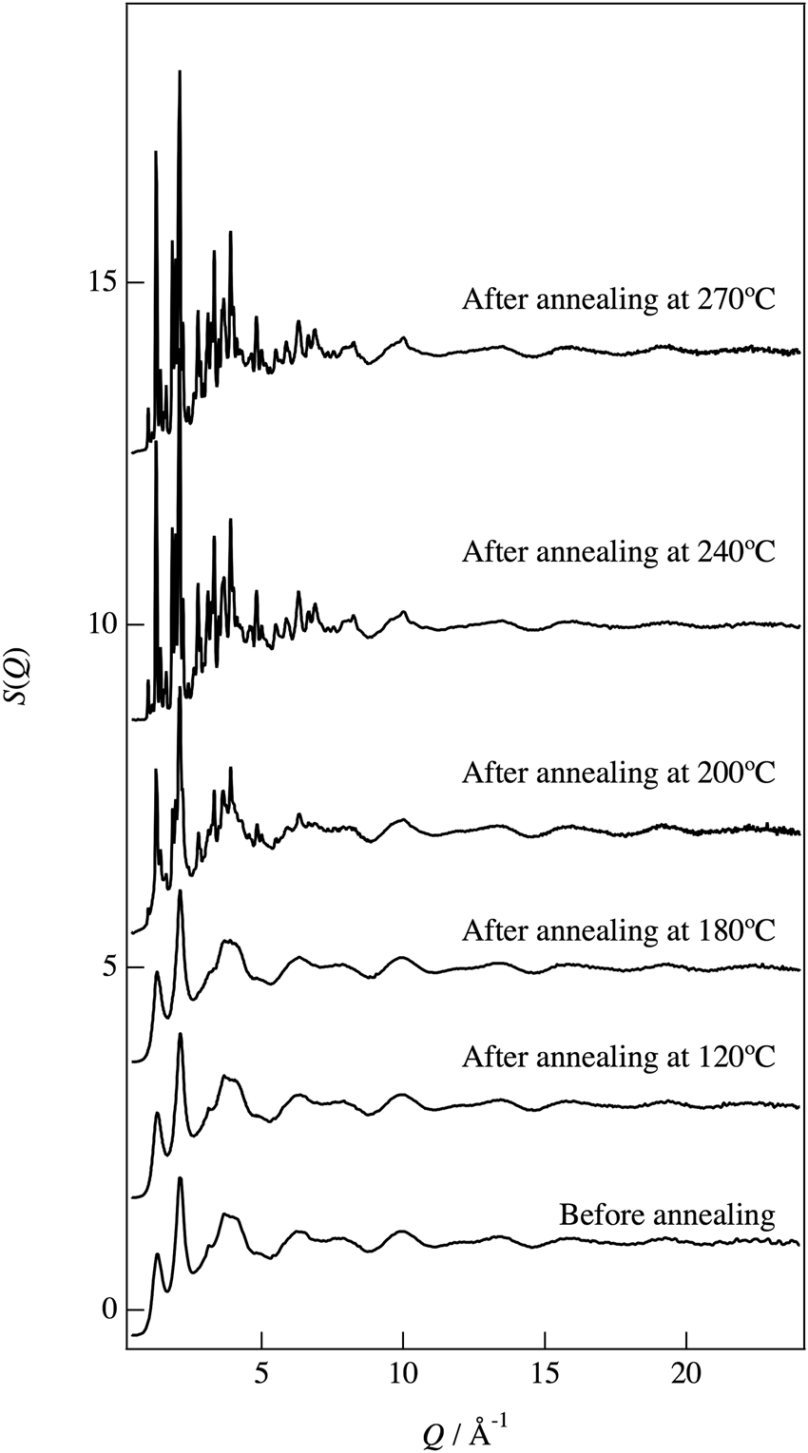
Total structure factors, *S*(*Q*), for the 75Li_2_S-25P_2_S_5_ glasses obtained from X-ray diffraction before annealing and after annealing at 120 °C, 180 °C, 200 °C, 240 °C, and 270 °C.

The 75Li_2_S-25P_2_S_5_ glasses examined in this study were prepared by mechanical milling, which induced results of an endothermic change due to a glass transition at around 209.3 °C according to the differential thermal analysis (DTA), as shown in Fig. 1. A sharp exothermic peak due to crystallization was observed at around 229.3° C in the DTA curve. Figure 2 shows the experimental X-ray total structure factors for the 75Li_2_S-25P_2_S_5_ glasses under each annealing condition. Bragg peaks corresponding to the crystalline phases are observed at 200 °C, 240 °C, and 270 °C. Oscillations in both the glass and crystalline phases remain up to the high values of *Q*, which is evidence for well-defined short-range order in the formation of P-S bonds. To extract quantitative information on the atomic arrangements in both phases from the diffraction, the reduced PDF, *G*(*r*), were calculated for each annealing condition by Fourier transformation of the total structure factor as follows:

1 $$G(r)=\frac{2}{\pi }{\int }_{{Q}_{min}}^{{Q}_{max}}Q\{S(Q)-\mathrm{1\}}{\rm{s}}{\rm{i}}{\rm{n}}(Qr)dQ\mathrm{.}$$

**Figure 3:**
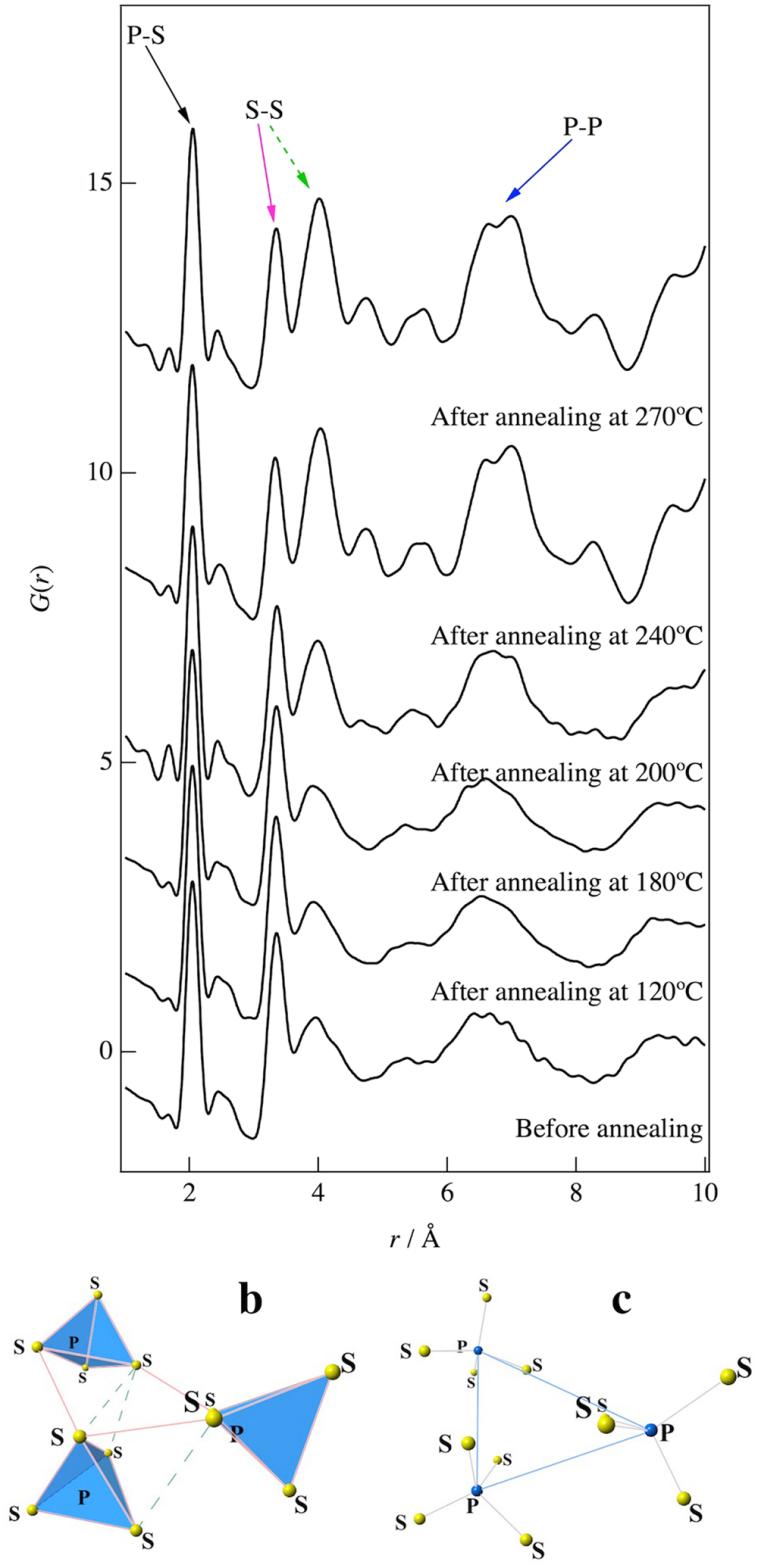
Reduced pair distribution functions, *G*(*r*), obtained from a Fourier transformation of *S*(*Q*) and Atomic configurations for the 75Li_2_S-25P_2_S_5_ glasses: (**a**) *G*(*r*) before annealing and after annealing at 120 °C, 180 °C, 200 °C, 240 °C, and 270 °C. (**b**) Typical atomic configuration of S-S correlations. The correlations at around 3.3 Å and 4.1 Å are connected by pink and green lines, respectively. The PS_4_ tetrahedral anions are highlighted by blue. (**c**) Typical atomic configuration of P-P correlations. The correlations at around 7.0 Å are connected by blue lines.

Figure 3 shows *G*(*r*) for each annealing treatment. On the basis of a previous study^10^, the first peak at around 2.0 Å is related to the P-S correlation associated with the PS_4_ tetrahedral anions and has no dependence on the annealing treatment. This means that the PS_4_ tetrahedral anions remain in the structure during the crystallization. On the other hand, the relative intensities of the second and third peaks at around 3.3 Å and 4.1 Å, which are related to the S-S correlations as shown in Fig. 3b, change between the glassy and crystalline phases. The second peak decreases upon crystallization, whereas the third peak increases. The peak at around 7.0 Å is related to the P-P correlation as shown in Fig. 3c and increases upon crystallization, which indicates the formation of ordered PS_4_ tetrahedral anions.

PDF analysis is a long-established technique for studying the local structure of amorphous and disordered crystalline materials^11, 12^. To clarify the amorphous and crystalline phase fractions in the two coexisting phases, the differential PDF (d-PDF) method was used^13, 14^. In the methodology, we can assume that *G*_*glass−ceramic*_ (*r*) can be estimated using the following equation:

2 $${G}_{glass-ceramic}(r)=\mathrm{(1}-x){G}_{glass}(r)+x{G}_{crystal}(r),$$

where *G*_*glass-ceramic*_ (*r*) is the measured PDF of a mixed sample, and *x* is the fraction of the crystalline component; *x* denotes the crystallinity. The sulfide glass is almost completely the crystalline phase after annealing at 270 °C. The glassy phase of interest after annealing at 200 °C was extracted by the d-PDF technique using the following equation:

3 $${G}_{g-{200}^{^\circ }C}\,(r)=\{{G}_{gc-{200}^{^\circ }C}(r)-x{G}_{c-{270}^{^\circ }C}\,(r)\}\mathrm{\}/(1}-x\mathrm{).}$$

**Figure 4:**
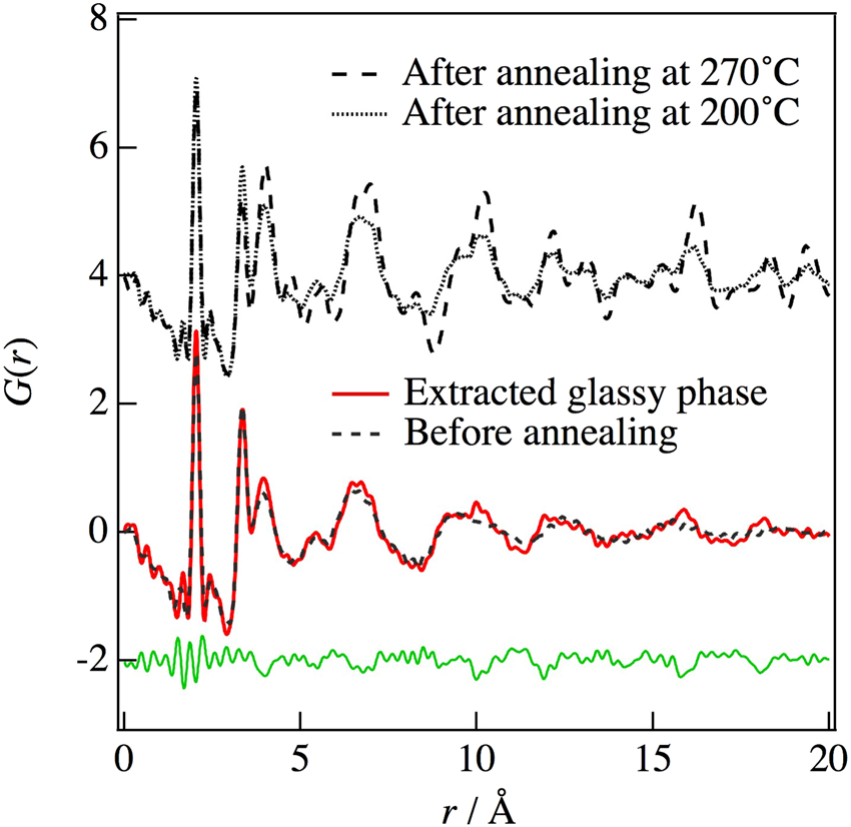
Comparison between the PDFs determined by equation (3) using the differential pair distribution functions: upper dashed line, after annealing at 270 °C; dotted line, after annealing at 200 °C; lower dashed line, before annealing; red solid line, glassy phase extracted for annealing at 200 °C.

**Figure 5:**
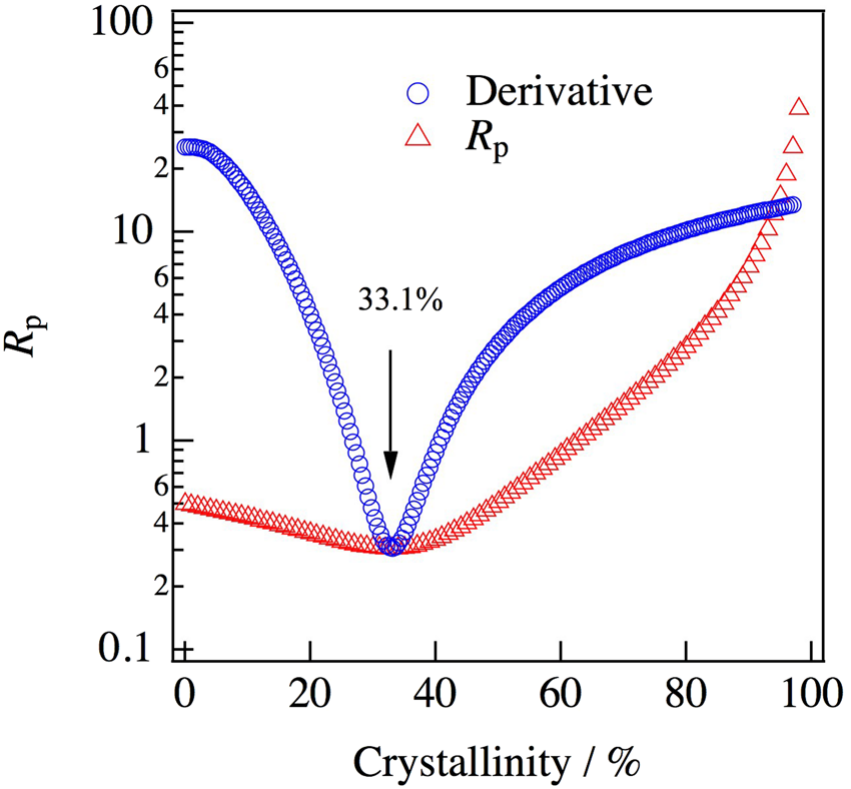
Reliability factors, *R*_*p*_, for the extracted glassy phase based on the crystallinity: red triangles, *R*_*p*_; blue circles, derivative of *R*_*p*_.

**Figure 6:**
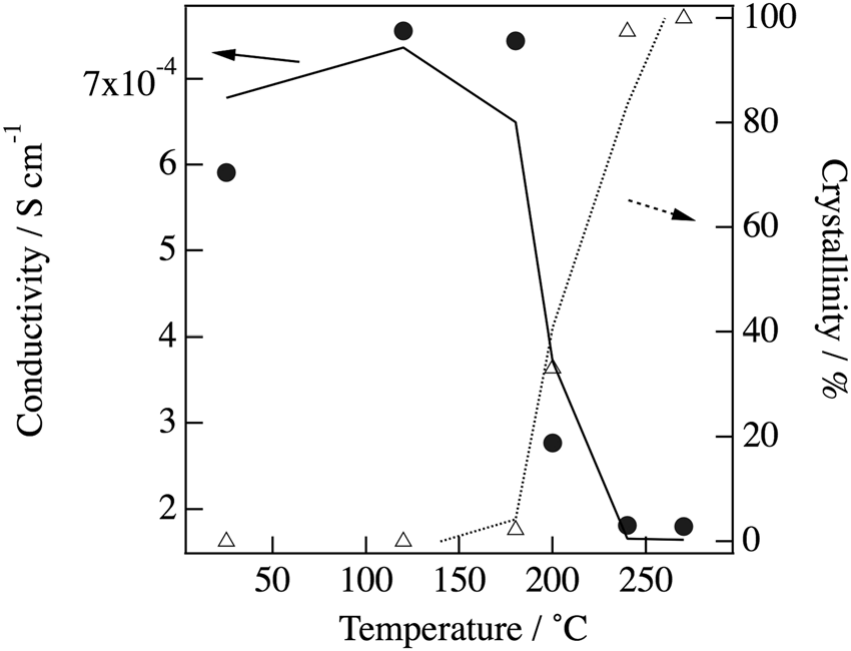
Crystallinity obtained from d-PDF under each annealing condition and the lithium ionic conductivity.

**Figure 7:**
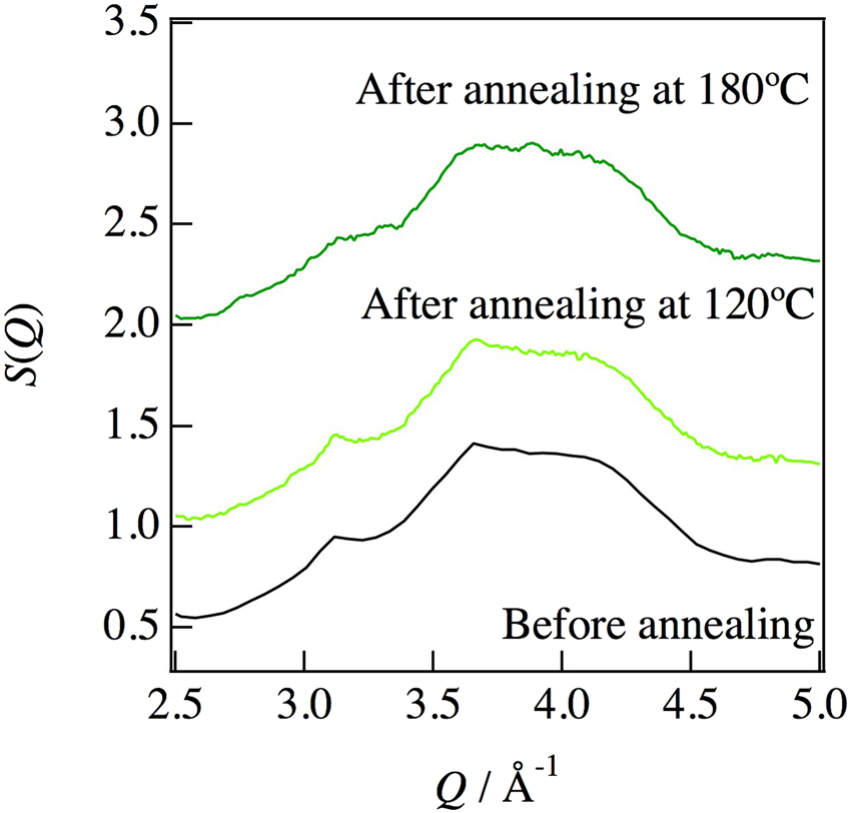
Structure factors in the range of 2.5–5.0 Å^−1^ from Fig. 2 enlarged for clarity.

**Figure 8:**
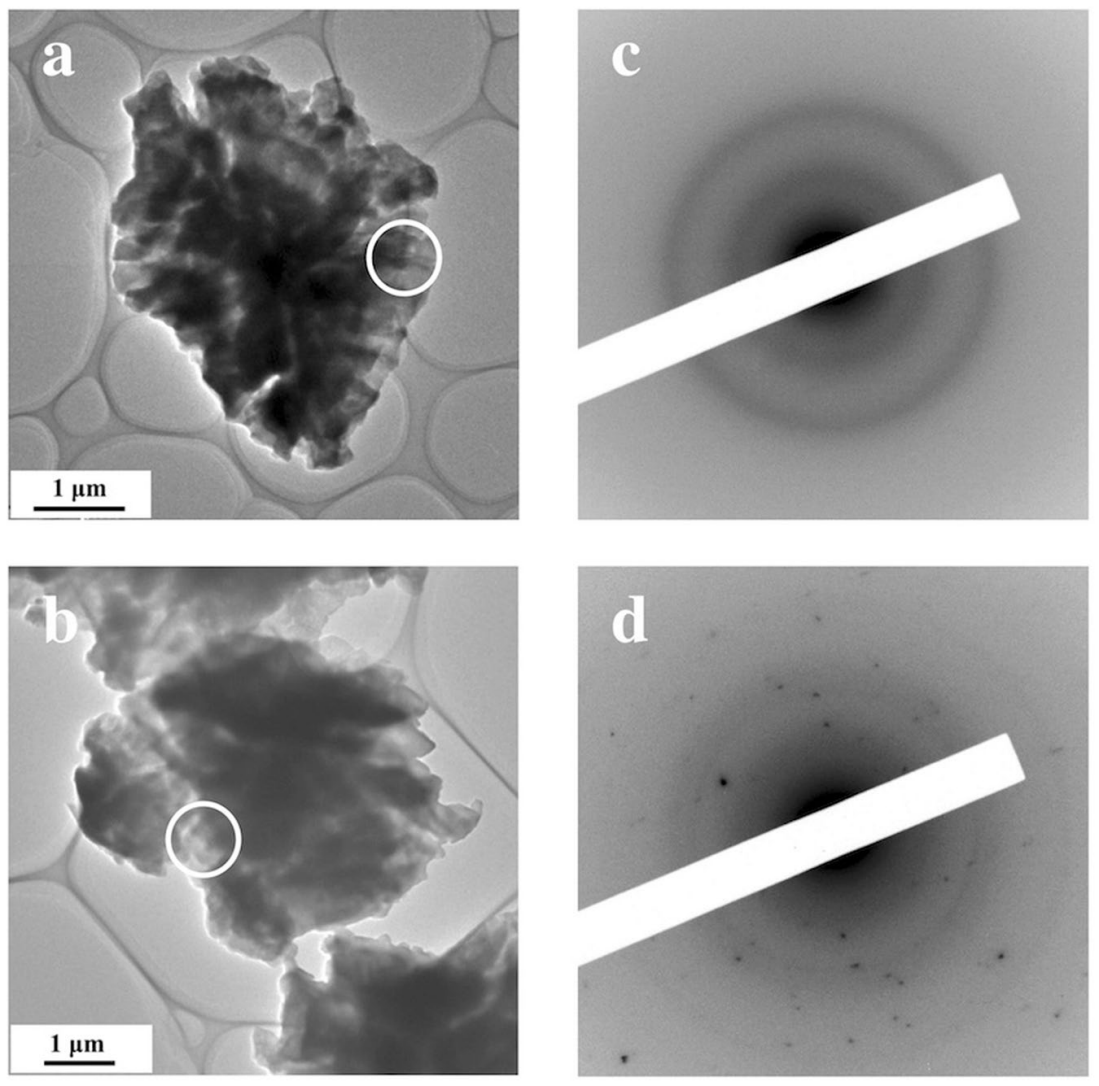
TEM images and selected area electron diffraction patterns of the 75Li_2_S-25P_2_S_5_ sulfide glass ceramic. (**a**,**b**) Bright-field images before annealing and after annealing at 180 °C. (**c**,**d**) Selected area electron diffraction patterns taken from the bright-field images.

Figure 4 shows the PDFs obtained before annealing and after annealing at 200 °C and 270 °C, as well as that of the extracted glassy phase at 200 °C for *r* = 0 to 20 Å. A slight difference in the PDF between the glassy phase after annealing at 200 °C and that before annealing was observed at *r* = 4.1 Å, 10 Å, and 16 Å. Peterson *et al*.^13^. mentioned that when utilizing this methodology, it is important to normalize data on an absolute scale and to have accurate models or standard data sets for the phases of interest. In this study, the standard data set is that before annealing the glassy phase. The pattern reliability factor (*R*-factor) of the d-PDF was *R*_*p*_ = 30.9% for *x*_*NMR*_  = 30.4%. From the peak deconvolution of ^31^P NMR spectra^15^, the crystallinity *x*_*NMR*_ was calculated. As mentioned above, the peak at *r* = 4.1 Å is related to the order of the crystalline phase. Here, we determined the crystallinity *x* to eliminate the peak difference at *r* = 4.1 Å, which indicated that *R*_*p*_  = 37.2% for *x* = 43.0%. Since the crystallinity is not consistent with the result of NMR, we calculated the correlation between *R*_*p*_ and the crystallinity by the d-PDF technique as shown in Fig. 5. The minimum value of *R*_*p*_ was estimated by logarithmic fitting and is shown as open circles in Fig. 5. It indicates that *R*_*p*_  = 30.5% for *x*_*PDF*_  = 33.1%. Figure 6 summarizes the crystallinity under each annealing condition. As can be seen in this figure, the ionic conductivity decreased after crystallization. It is well known that glasses have long-range density fluctuations in their structures^16, 17^. Such fluctuations may be related to the improvement of ionic conductivity by the structural relaxation caused by the annealing treatment. However, the d-PDF indicated that the glassy structure shows no structural change in the sulfide glass-ceramic electrolytes. On the other hand, Schirmeisen *et al*. suggested that the fast ionic conduction of mobile ions in nanostructured LiAlSiO_4_ glass ceramics is caused by the local movement of ions at the interfaces between the glassy phase and embedded crystallites. The existence of an electrical relaxation process with a very low activation energy, which is absent in pure LiAlSiO_4_ glass, has been demonstrated by time-domain electrostatic force spectroscopy^18^. Actually, we observed the formation of a nanocrystalline phase in the diffraction pattern after the annealing, which means that the Bragg peaks were deformed, as shown in Fig. 7. Moreover, the nanocrystalline phase was verified by TEM and electron diffraction. Figure 8a and b show bright-field (BF) images of sulfied glassy electrolytes before annealing and after annealing at 180 °C, respectively. The electron diffraction patterns obtained from the BF images are shown in Fig. 8c and d. Spots can be seen in the electron diffraction after annealing as compared with that before annealing by this preliminary analysis. Thus, we suggest that the existence of such nanocrystalline phases, which are a minority component in the glass ceramic, will lead to an increase in the ionic conduction as compared with that of a pure sulfide glass.

## Conclusion

The 75Li_2_S-25P_2_S_5_ glass showed a higher conductivity of 7.5 × 10^−4^ S cm^−1^ as a result of annealing before the crystallization, and a lower value of 2.0 × 10^−4^ S cm^−1^ after the crystallization. The differential pair distribution function was utilized as a methodology for the mixed materials in this study to extract the glassy structure in the 75Li_2_S-25P_2_S_5_ sulfide glass ceramic. This method quantitatively reproduced the fraction of mixed phases, which was in agreement with the result of NMR. The extracted glassy structure exhibited no changes during the annealing treatment. Owing to the crystallization of the majority phase, the ionic conductivity in this glass decreased after crystallization. We observed the formation of a nanocrystalline phase in the X-ray and electron diffraction patterns obtained during the annealing. Thus, our present finding of minority component is expected to lead to further understanding for the development of solid electrolytes with high ionic conductivity.

## Methods

### AC impedance measurement

Ionic conductivity was measured by the AC impedance method in an Ar atmosphere at room temperature with an applied frequency range of 0.1 Hz to 1 MHz using a Solartron 1260 frequency response analyzer. Carbon-coated blocking the electrode was painted on both sides of the sample. The observed ionic conductivities before annealing and after annealing at 120° C, 180° C, 200° C, 240° C, and 270° C were 5.9 × 10^−4^ S/cm, 7.6 × 10^−4^ S/cm, 7.4 × 10 ^−4^ S/cm, 2.8 × 10^−4^ S/cm, 1.8 × 10^−4^ S/cm, and 1.8×10^−4^ S/cm, respectively.

### High-energy X-ray diffraction measurement

The high-energy X-ray diffraction experiments for the 75Li_2_S-25P_2_S_5_ glasses were carried out at room tempera-ture at the SPring-8 high-energy XRD beamline BL04B2 using a two-axis diffractometer^19^. The incident X-ray energy obtained from a Si 220 crystal monochromator was 61.4 keV. The diffraction patterns of the samples and an empty tube were measured in the transmission geometry with an angle from 0.3 to 48°, corresponding to a *Q*-range from 0.2 to 25 Å^−1^. The intensity of the incident X-ray was monitored in an ionization chamber filled with Ar gas and the scattered X-rays were detected by CdTe detectors. A vacuum electric chamber was used to suppress air scattering around the sample. The collected datasets were corrected for the absorption, background, and polarization effects.

### TEM measurement

To obatain bright-field (BF) images and electron diffraction (ED) patterns, TEM observations were carried out using JEM-2100F field-emission-type TEM systems. Samples were mounted on an amorphous carbon film supported by a Cu grid for TEM observation, which was then attached to a TEM vacuum holder in a glove box filled with an Ar gas. The vacuum degree was approximately 1.0 × 10^−5^ Pa.
